# Time models and cognitive processes: a review

**DOI:** 10.3389/fnbot.2014.00007

**Published:** 2014-02-27

**Authors:** Michail Maniadakis, Panos Trahanias

**Affiliations:** Institute of Computer Science, Foundation for Research and Technology - HellasHeraklion, Greece

**Keywords:** artificial sense of time, temporal cognition, literature review, computational modeling, time perception

## Abstract

The sense of time is an essential capacity of humans, with a major role in many of the cognitive processes expressed in our daily lifes. So far, in cognitive science and robotics research, mental capacities have been investigated in a theoretical and modeling framework that largely neglects the flow of time. Only recently there has been a rather limited, but constantly increasing interest in the temporal aspects of cognition, integrating time into a range of different models of perceptuo-motor capacities. The current paper aims to review existing works in the field and suggest directions for fruitful future work. This is particularly important for the newly developed field of artificial temporal cognition that is expected to significantly contribute in the development of sophisticated artificial agents seamlessly integrated into human societies.

## 1. Introduction

The sense of time is an essential capacity of humans, animals, birds, fishes, even plants (Cashmore, 2003). Time perception is among the first competencies evolved in biological systems and thus, it has affected the subsequent evolution of nearly all cognitive modalities (Paranjpe and Sharma, 2005; Gerstner, 2012). Therefore, it is really surprising that many time-dependent cognitive capacities have traditionally been investigated in a theoretical and modeling framework that largely neglects the flow of time (for years, we have studied a timeless memory, attention, action planning, etc.). Only recently, limited research efforts were addressed to the temporal aspects of cognition, integrating time into a range of perceptual and motor skills (Taatgen et al., 2007; Battelli et al., 2008; Holcombe, 2013).

Moreover, sense of time and temporal cognition are largely missing from robotic systems, with a clear negative impact in the integration of autonomous artificial agents into human environments. This is because the core idea of symbiotic human-robot interaction assumes the close, synchronized and temporally extensive coupling between humans and machines. However, existing systems assume short-term and nearly momentary interaction between the two ends, largely ignoring the inherent temporal dimension of human-robot synergism. Recent studies have revealed time as an entity that can be processed in its own right by artificial autonomous systems (Maniadakis et al., 2009; Maniadakis and Trahanias, 2013).

This article reviews computational modeling works that consider the inherent temporal characteristics of cognition and highlights the great potential of equipping robotic systems with time processing capacities. The review mainly focuses on efforts considering how the perception of time can be used in conjunction with other cognitive or behavioral skills. Therefore, despite the fact that a large number of computational models have been introduced in the last years to explain time perception mechanisms in the brain, they are not considered to be in the center of this review and will be discussed very briefly. The interesting reader is suggested to consider recent review papers in this direction (Wittmann, 2013).

The article is structured as follows. A brief review of neurophysiological findings on time perception is first attempted and the main types of computational models of interval timing are discussed. Following that, computational models addressing the interaction of time perception with other cognitive skills are reviewed. Finally, the vital role of sense of time in human-centered naturalistic human-robot interaction is addressed and directions for fruitful future research are suggested.

## 2. Time processing mechanisms in the brain

Over the past decade, a number of different brain areas have been implicated as key parts of a neural time-keeping mechanism in the milliseconds-to-a-few-seconds time range and discussed together with assumed functional properties: notably (among many others), event timing in the cerebellum (Ivry and Spencer, 2004), generalized magnitude processing for time, space and number in the right posterior parietal cortex (Bueti et al., 2008; Oliveri et al., 2009), working memory related integration in the right prefrontal cortex (Lewis and Miall, 2003; Smith et al., 2003), a right fronto-parietal network (Harrington et al., 1998), coincidence detection mechanisms using oscillatory signals in fronto-striatal circuits (Hinton and Meck, 2004), hippocampal time-cells focused on the relation of time and distance (Kraus et al., 2013), as well as integration of ascending interoceptive (that is, body) signals in the insular cortex (Craig, 2009; Wittmann, 2009).

The participation of many brain areas in the processing of temporal information attest the key role of time in multiple aspects of cognition such as decision making, action planning, memory storage and recall, etc. (Rao et al., 2001; Livesey et al., 2007; Taatgen et al., 2007).

## 3. Computational models of time perception

In an attempt to explain where and how time is processed in the brain, a large number of neurocomputational models have been implemented, most of them concentrating on duration perception. Broadly speaking, two main approaches have been proposed in the literature to describe how our brain represents time (Ivry and Schlerf, 2008; Bueti, 2011). The first is the dedicated approach (also known as extrinsic, or centralized) that assumes an explicit metric of time. This is the oldest and most influential explanation on interval timing. The models included in this category employ mechanisms that are designed specifically to represent duration. Traditionally such models follow an information processing perspective in which pulses that are emitted regularly by a pacemaker are temporally stored in an accumulator, similar to a clock (Woodrow, 1930; Gibbon et al., 1984; Droit-Volet et al., 2007). This has inspired the subsequent pacemaker approach that uses oscillations to represent clock ticks (Miall, 1989; Large, 2008). Other dedicated models assume monotonous increasing or decreasing processes to encode elapsed time (Staddon and Higa, 1999; Simen et al., 2011). The second approach includes intrinsic explanations (also known as distributed) that describe time as a general and inherent property of neural dynamics (Dragoi et al., 2003; Wackermann and Ehm, 2006; Karmarkar and Buonomano, 2007). According to this approach, time is intrinsically encoded in the activity of general purpose networks of neurons. Therefore, rather than using a time-dedicated neural circuit, time coexists with the representation and processing of other external stimuli. However, besides the key assumption of multi-modal neural activity, the existing computational implementations of intrinsic interval timing models are not yet coupled with other cognitive or behavioral capacities within a broader functional context, and in that sense, the internal clock remains unaffected by outside processes. Only the Behavioral Theory of Timing (Killeen and Fetterman, 1988) and the Learning to Time (Machado, 1997) make explicit coupling between time perception and behavior, assuming that the behavioral vocabulary of subjects and their current behavioral state support duration perception.

The main limitation of the dedicated approach regards its weakness in explaining modality specific differences in time perception. On the other side, intrinsic models are considered to have limited processing capacity, therefore considered inappropriate to accomplish duration processing in complex and real life tasks. However, both modeling approaches are supported by neurophysiological and behavioral observations and the debate concerning the representation of time in the brain is now more active than ever.

An attempt to combine the two approaches is provided by the Striatal Beat Frequency (SBF) model which assumes that timing is based on the coincidental activation of basal ganglia neurons by cortical neural oscillators (Matell and Meck, 2004; Meck et al., 2008). The SBF model assumes a dedicated timing mechanism in the basal ganglia that is based on monitoring distributed neural activity in the cortex. Recently, SBF has been integrated into a generalized model of temporal cognition that subserves different aspects of perceptual timing, either duration based or beat-based (Teki et al., 2012).

## 4. Cognitive models exploiting sense of time

Despite the essential role of temporal cognition in the survival and social organization of humans and animals, a surprisingly small number of computational models have been implemented that address the integration of sense of time with other cognitive modalities. This section provides an outline of the existing computational models discussing the topics addressed so far, in an attempt to reveal the broad range of cognitive processes directly associated with the perception of time.

We note that, being capable to experience and process time is drastically different to what is now known as the dynamic cognition approach (Van Gelder, 1998; Beer, 2000). The latter considers brain as a dynamical system that is strongly linked with the body and the continuously changing environment. Previous works have examined tasks that involve spatio-temporal characteristics, such as self-localization by means of information integration over time (de Croon et al., 2006), and turn-taking alternation to coordinate the behavior of two agents (Iizuka and Ikegami, 2004). However, despite considering the coupled spatio-temporal nature of real world phenomena, the dynamic cognition approach has mainly focused on information integration over time and has not provided cognitive systems with any kind of time perception that is valid in its own right, or sense of time that is amenable to processing.

In contrast to the above, the focus of the present article is on works with a clear and explicit reference to the notion of time *per se*. The description of existing works will concentrate on the qualitative characteristics of time interaction with numerous cognitive modalities, avoiding implementation details. The works are listed below.

### 4.1. Time in decision making

The explicit perception of the notion of time by robotic agents is first witnessed in a study on self-organized robotic cognitive system (Maniadakis et al., 2009). Similar to animals that are capable of learning the temporal structure of tasks, artificial agents that have been evolved to accomplish a behavioral rule switching task that resembles Wisconsin Card Sorting, distinguish the available rules by considering the temporal properties of their own behaviors. This time-perception mechanism that has emerged in the robot's brain without being requested by the designers of the model suggests that the equipment of artificial agents with sense of time may significantly enhance their cognitive capacities.

### 4.2. A grounded temporal lexicon

The exploration of how robots can be aware of the temporal aspects of events is discussed in Schulz et al. (2011). Lingodroids (language learning robots) have been used to learn terms for space and time. Cognitive maps constructed by individual agents from their own journey experiences have been used for grounding temporal concepts in robot languages to answer the question how long did it take to complete a journey. In a series of studies, the authors demonstrated that a spatio-temporal lexicon for journey duration can be grounded, that is linked to the state of the world, using a variety of concepts. Effective concepts and names for duration provide a first step toward a grounded lexicon for temporal intervals. Even if spatial and temporal terms are not identical, the study showed that both can be learned using similar language evolution methods, and that time, distance, and change can serve as proxies for each other under noisy conditions.

### 4.3. Interval timing grounded in motor activity

This work is relevant to the category of time perception models that assume memory-trace decay. Such models can not associate memory-trace decay into temporal information, unless there is a means of grounding that decay in meaningful, repeatable event sequences. Infant motor activity is a plausible way in which this calibration could be achieved early in development as suggested in Addyman et al. (2011). In other words, body and arm movement serve as a rough temporal yardstick for visual and auditory memory-trace decay. The implemented model learns an association between limb movement and how long ago an event took place (as measured by activation decay of a memory trace). Therefore, embodied learning leads to the calibration and synchronization of clocks in audition and vision metronome for the timing of memory decay.

### 4.4. Representation of duration

In an attempt to perform an unconstrained investigation of the possible representations of duration in cognitive systems, self-organized computational models can serve as a new tool that facilitates the exploration of alternative representations. In Maniadakis and Trahanias (2013) a single robotic cognitive system is employed to accomplish two different robotic behavioral tasks which assume diverse manipulation of time intervals. The careful examination of the artificial brains puts forward a new representation of time that incorporates characteristics from both the “dedicated” time representation approach and the “intrinsic” time representation approach (see section 3).

### 4.5. Time perception as a secondary task

The incorporation of a timing module in the ACT-R architecture enables the investigation of how the timing mechanisms interact with other aspects of cognition (Taatgen et al., 2007). The basis of the module is a pacemaker-based internal clock. Interaction of the clock module with the rest of the ACT-R system allows explanations for the role of timing, attention, perception and learning in the accomplishment of complex tasks. An exemplar scenario regards the exploration of time perception in contexts where interval timing itself is secondary to a main task, since this is often the natural role time estimation plays in everyday life. By investigating a complex task in which keeping track of time intervals is only a single aspect of what participants have to do, the composite model provides an abstracted view on how sense of time interacts with a complex set of cognitive processes and highlights the key role of time in large scale brain functioning.

### 4.6. Past, future perception

The evolution of perceptual capacities beyond the here and now of the world are discussed in Choe et al. (2012). The conditions that have facilitated the emergence of the notion of past and future in the form of memory and prediction, respectively, are investigated through the simulated evolution of simple neural networks. Memory has been evolved as an autonomous self-driven mechanisms that is significantly more powerful compared to stigmergy (i.e., the production of a certain behavior in agents as a consequence of the effects produced in the local environment by previous behavior). Moreover, predictable internal states dynamics turned out to have a high selective value in evolution, resulting in significantly more robust systems, compared to equally performing memory-less systems which develop much more fragile internal mechanisms.

### 4.7. Mental time travel

Our ability to recall the past or imagine the future is referred with the term Mental Time Travel. Capitalizing on extensive neurophysiological and computational modeling studies on the functionality of hippocampus, it is possible to construct a computational model that explains the ability to recall and potentially re-experience a previously experienced event, by associating specific stimuli with specific memories (Hasselmo, 2009). The implemented brain-like model uses representations of head direction activity, entorhinal grid cell activity and hippocampal place cell activity to perform encoding and retrieval of episodic memory. The model has so-far addressed recalling trajectories experienced as continuous curves through space and time, but it can be extended to describe more complex events.

### 4.8. Learning through time

The role of time in conditional learning is discussed in Howard (2014). Considering the Stimulus Sampling Theory, one of the first rigorous mathematical models for learning that describes how the memory representation of stimulus changes over time, it is possible to explore the temporal properties of learning. The paper contrasts subsequent mathematical models of learning to SST and associates neuroscientific data of brain activity at different times, with predictions from mathematical models describing cognition.

### 4.9. Forgetting

The typical explanation of forgetting assumes information to decay over time making information held in short-term memory to be quickly forgotten unless it is constantly rehearsed or refreshed. The Time-Based-Resource-Sharing (TBRS) is one of the most successful explanations addressing the intricate trade-off between deterioration and restoration of memory. A computational instantiation of the TBRS, is presented in Oberauer and Lewandowsky (2011) explaining how working memory evolves and reshapes through time. The model accomplishes to successfully explain behavioral data on particularly complex working memory tasks.

### 4.10. Memory reconsolidation

According to the idea of reconsolidation, the retrieval of memories returns their representation to a plastic state, which means that the memory can be changed or even erased. In Sederberg et al. (2011) an existing computational model of memory retrieval, the Temporal Context Model (TCM) is employed to explain human reconsolidation data. Episodic encoding in TCM involves binding items to their temporal encoding context. Retrieval involves cueing with a temporal context, which then reinstates the memory item through a learning procedure. Computational experiments show that TCM successfully addresses short-term and long-term recency and contiguity effects in memory encoding and manipulation.

## 5. Discussion

**Figure 1:**
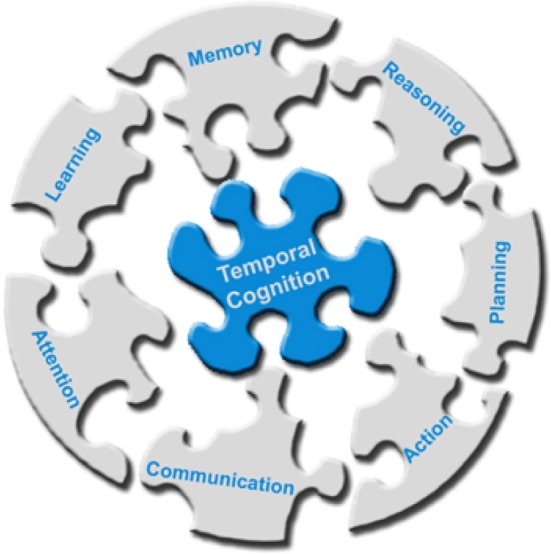
**Temporal cognition operates as the cognitive glue that integrates cognitive skills in order to effectively accomplishing high-level intelligence**.

The works listed above provide evidence for the multimodal interaction between sense of time and cognition that goes far beyond the perception of duration, addressing a very broad range of cognitive functions. This suggests temporal cognition as the cognitive glue that enables the integration of skills into a coherent smoothly functioning composite system (Figure 1). Along this line, we have previously argued that a key milestone for the development of truly autonomous and intelligent artificial agents regards implementing computational systems capable of considering the temporal aspects of cognition (Maniadakis and Trahanias, 2011).

In addition to the frequent argument that “time is ever present in life” (Mittelstrass, 2001) the famous philosopher Immanuel Kant (1724–1804) has described time as “… an unavoidable *framework* of the human mind that preconditions possible experience”. According to this view, cognition is shaped by the temporal properties of the world and our ability to engage them in our thoughts.

Consider for example the case of taking a decision under time pressure. The choice that we make emerges from the requirement of a fast decision, and if this temporal constraint was absent a completely different decision would probably be made. In a different context, when we discuss with friends trying to recall a specific event from high-school, a part of our mind is situated into the past high-school period, while another part of our mind remains focused into the present, coordinating the real time communication with friends. This ability to simultaneously situate our mind in multiple periods in the past-present-future timeline is inherent in our brain, and is crucial for our daily activities. Therefore, our thoughts and experiences are not only situated in space but also in time, suggesting an innate *entimed* nature for cognition that nicely complements the well known embodied nature of cognition. While embodiment focuses mainly on the here and now of the world, entiment additionally postulates that past experiences and future goals beyond the here and now are also very important for the brain to develop cognition. Interestingly, the concepts of entimed and embodied cognition bridge when we consider the temporal properties of the “present,” an abstract term that is very flexibly defined by humans to include moments of both the past and the future (e.g., I am writing a book, now). To sufficiently perceive the current state of the world and understand our role in it, we have to consider both the spatial and the temporal properties of the environment. In other words, our minds are situated both in space and time. If either the space or the time is differentiated, then the state of our mind will also be differentiated.

Turning into the machinery of the cognitive systems, we can identify two broad aspects on how time affects cognition:

- Implicitly, as a key regulator of internal cognitive mechanisms. Time determines our ability to learn and forget (Oberauer and Lewandowsky, 2011; Howard, 2014), the temporal allocation of mental resources (Salvucci and Taatgen, 2008), the effect of habituation on cognition (Rankin et al., 2009), the sense of self (Zeman and Coebergh, 2013), and our whole perspective on life (Zimbardo and Boyd, 1999).
- Explicitly, as a sensory modality or information that can be processed in its own right. Time can be perceived and processed (Wittmann, 2013), be abstracted quantitatively (Dehaene and Brannon, 2010) or qualitatively (Silva et al., 2007), passed as a parameter from one cognitive process to another (Miles et al., 2010), support perceptuo-motor activities in the 4D rather than 3D world (Wallis, 1967).

Unfortunately, in the field of artificial systems, even state of the art devices cannot handle time in a way comparative to humans. As devices and systems are becoming increasingly powerful, the typical momentary interaction between humans and computers is often lagging behind and constitutes a bottleneck for fully exploiting the power of modern devices. Moreover, in the field of robotics, research has mainly focused on technical problems related to the accomplishment of complex behavioral tasks, but has not paid sufficient attention to temporal cognition and its vital role in implementing systems that live for long periods in the real world. Unfortunately, this enhances the phenomenon of “uncanny valley” that concerns the acceptance of robots as intelligent artificial partners. The more a robot is made to resemble a human, the more sensitive humans become to the subtle cognitive differences, shattering the illusion of intelligence. Therefore, the lack of temporal cognition in robotics acts as an obstacle in their symbiosis with humans.

Next generation robotic cognitive systems are necessary to offer new modes of interaction that will base on the deep understanding of the long-term trajectories of human machine confluence (see Box 1). Even if humans have conventionally structured their lifetime into past, present and future, artificial systems have not so far developed these notions in an adequate level, therefore being unable to understand and adapt to the heavily time-structured human social life. To proceed effectively toward implementing artificial temporal cognition, it is necessary to consider the natural, developmental procedure of the human brain that enables different time processing capacities to develop and integrate with other cognitive skills. While primary sense of time capacities mature very early in the human developmental procedure, our temporal cognition skills continuously improve until adolescence (Droit-Volet, 2011; Tucholska and Gulla, 2012). Following a similar incremental procedure, computational implementations should first focus on basic skills such as duration processing or synchrony, then consider the wider timeline that spans over past present and future to explore time in memory, attention, learning, and action planning, proceed with time language interactions and finally consider how time integrates into complex cognitive capacities such as mind reading, or imagination. Future works along these unpaved pathways are expected to have high impact in developing the next generation of autonomous intelligent systems.

Box 1 **Outstanding problems in computational temporal cognition**

- Where is the source of temporal sensory experiences in the human and animal brain and what this suggests for the computational representation of time?
- How can we computationally represent the property of unidirectional temporal flow that separates time from all other perceptual dimensions?
- How we perceive different temporal granularities and how we assign time a numerical meaning?
- What is the best way to smoothly integrate time processing capacities in the existing (time-lacking) cognitive systems?

## 6. Conclusions

Time is ubiquitous in brain functioning and cognition. The present article presents early attempts linking cognitive capacities with numerous aspects of sense of time and temporal cognition. The largely heterogenous approaches adopted so far, render the integration of the models and the development of a single composite system a very challenging future research topic. However, making progress toward the development of a unified system capable to consider the flow of time is expected to provide new impetus in symbiotic human–machine interaction.

Clearly, if we are going to ever implement intelligent robots that live next to us and operate in a way comparable to humans, then these robots will be definitely equipped with advanced time perception and processing capacities.

### Conflict of interest statement

The authors declare that the research was conducted in the absence of any commercial or financial relationships that could be construed as a potential conflict of interest.
